# Epidemic, risk factors of carbapenem-resistant *Klebsiella pneumoniae* infection and its effect on the early prognosis of liver transplantation

**DOI:** 10.3389/fcimb.2022.976408

**Published:** 2022-10-06

**Authors:** Ning Liu, Gengxia Yang, Yan Dang, Xin Liu, Ming Chen, Fangfang Dai, Xiurong Ding, Wenlei Li, Guangming Li, Jinli Lou, Dexi Chen, Yanhua Yu

**Affiliations:** ^1^ Department of Clinical Laboratory Center, Beijing Youan Hospital, Capital Medical University, Beijing, China; ^2^ Beijing Institute of Hepatology, Beijing Engineering Research Center for Precision Medicine and Transformation of Hepatitis and Liver Cancer, Beijing Youan Hospital, Capital Medical University, Beijing, China; ^3^ Department of General Surgery, Liver Transplantation Center, Beijing Youan Hospital, Capital Medical University, Beijing, China

**Keywords:** liver transplantation, carbapenem-resistant *Klebsiella pneumoniae*, epidemic, mortality, risk factors

## Abstract

**Background:**

Carbapenem-resistant *Klebsiella pneumoniae* (CRKP) infection remains a major cause of morbidity and mortality in early-stage post-liver transplantation (LT).

**Methods:**

We retrospectively analyzed the demographic and clinical infections characteristics of all LT recipients in our hospital between January 2019 and December 2021.

**Results:**

Among the 272 LT recipients who received LT between January 2019 and December 2021, sixty-two patients had at least one infection within 3-months post-LT, with a prevalence of 22.8% (62/272). The prevalence of CRKP infections was 7.0% (19/272), and the 3-months post-LT mortality was 19.4% (12/62). The risk factors independently related to 3-months mortality were age (Odds ratio (OR)= 1.126, 95% Confidence interval (CI): 1.009~1.257; P=0.034), mechanical ventilation (MV) (OR=1.206, 95% CI: 1.039~1.401; P =0.014), and CRKP infection (OR=18.240, 95% CI: 2.206~150.842; P =0.007). In CRKP infection, the length of ICU stay (OR=1.067, 95% CI: 1.015~1.122; P=0.011), pre-operation infection (POI) (OR=6.733, 95% CI: 1.160~39.088; P=0.034), and hepatocellular carcinoma (HCC) (OR=26.772, 95% CI: 1.747~410.187; P=0.018) were the independent risk factors. With COX multivariate regression analysis, the 3-months survival rate of CRKP infected patients was significantly lower than that without CRKP infection post-LT.

**Conclusions:**

CRKP infection is closely correlated with poor prognosis in 3-months post-LT.

## Background

Liver transplantation (LT) is the most effective treatment for end-stage liver disease. Infectious complications are major causes of morbidity and mortality in the early period post-LT, even the treatment and clinical care have dramatically improved (Fishman, 2007). Bacterial infections comprise the most frequent type of infection, followed by fungal, viral, and protozoal infections (Kim, 2014). Both gram-negative bacteria (GNB) (Chen et al., 2020; van Delden et al., 2020) and gram-positive bacteria (GPB) (Li et al., 2012; van Delden et al., 2020) are the major causative organisms consequent of epidemiological exposure. In addition, the application of antibiotics in the clinic may lead to drug-resistance bacteria. The acquisition of bacterial resistance ultimately affects mortality in LT recipients (Hand and Patel, 2016; Verma et al., 2022).

During the first three months post-LT, the patients are prone to opportunistic infections due to immunosuppression. Most infections are associated with surgical procedures and postoperative complications, including deep intra-abdominal infections, pneumonia, biliary infection, surgical site infection, bacteremia, urinary tract infections, and catheter-related infections (Romero and Razonable, 2011). The causative gram-positive cocci pathogens included *Enterococcus species*, *Staphylococcus aureus*, and *Dung enterococcus*. The causative gram-negative bacilli pathogens included *Acinetobacter baumannii*, *Klebsiella pneumoniae*, *Stenotrophomonas maltophilia*, *Pseudomonas aeruginosa*, *Escherichia coli*, and *Enterobacteriaceae* (Li et al., 2012). In recent years, the gram-negative bacilli have been shifted as the major pathogen of infection (Kim, 2014). The multi-drug-resistant (MDR) bacterial pathogens emerged with the clinical application of advanced antibiotics (Verma et al., 2022), such as Carbapenem-resistant *Klebsiella pneumoniae* (CRKP). Multiple studies have shown that the infection of MDR pathogens is the primary factor affecting the early survival rate of solid organ transplantation recipients (Li et al., 2014; Zhang et al., 2021).

In this study, we will investigate the epidemic of bacteria infection and the risk factors for mortality and CRKP infection in early 3-months post-LT.

## Materials and methods

### Study design and patient sample

The LT cases were recruited from Beijing Youan Hospital from January 2019 to December 2021. Three patients who died within 48 hours were excluded. Finally, a total of 272 cases were recruited. The endpoint of our study was the prevalence of CRKP infection and mortality within 3-months post LT. The LT recipients with bacteria infection within 3-months after LT were included in our study. The bacteria-infected patients were further divided into two subgroups, patients with CRKP infection and patients without CRKP infection.

All recipients were regularly followed up in the outpatient department post-LT. The recruited LT recipients received triple immunosuppression (tacrolimus, Prednisolone acetate or methylprednisolone, and mycophenolate mofetil). Rejection that does not respond to glucocorticoid therapy was treated with anti-human CD3 monoclonal antibody, anti-thymocyte immunoglobulin, or anti-lymphocyte immunoglobulin. Intraoperative prophylactic drugs and postoperative anti-infection treatment plan were third-generation cephalosporins (cefoperazone and sulbactam) or carbapenems (imipenem and meropenem). The antibiotics and dose were adjusted according to the infection situation and bacterial identification results. This study was approved by our institutional Ethics Review Committee (jingyoukelunyi (2021)020) and was conducted according to the Declaration of Helsinki principles.

### Clinical data collection

The patient’s clinical data were extracted from the electronic medical records (EMR), including demographics, infection characteristics, and other clinical variables. We investigated the epidemic of postoperative bacterial infections, clinical specimens, drug resistance, CRKP infection prevalence, and the three-months mortality. The risk factors for 3-months mortality were analyzed, including sex, age, diabetes mellitus (DM), hypertension (HTA), pre-operation infection (POI), hepatocellular carcinoma (HCC), liver failure (LF), multifocal infection, lung infection, CRKP infection, mechanical ventilation (MV), and length of intensive care unit (ICU) stay.

Only the first CRKP infection post LT was included for early CRKP infections. The variables were collected to assess the potential risk factors for CRKP infection, including sex, age, DM, HTA, POI, pre-operation disease (HCC, LF), multifocal infection, lung infection, length of MV, and length of ICU stay.

### Definition

Early infection was defined as an infection occurring in the first three months after liver transplantation. The standards used to define and classify infections in our study were proposed by the Centers for Disease Control and Prevention (Horan et al., 2008). The infection was defined based on combining the positive culture of different samples with the positive clinical manifestations. The samples were carried out for clinical bacteria culture, including blood, sputum (or other respiratory secretions), urine, ascites samples, organ preservation solution, catheter drainage fluid, and lavage fluid. CRKP was defined as insensitivity to at least one carbapenems, with a minimum inhibitory concentration ≥2 ug/mL for ertapenem or ≥4 ug/mL for imipenem or meropenem (Clinical and Laboratory Standards Institute, 2017).

### Microbiology

The clinical samples were collected for culture when any infection was suspected during the first three months of follow-up. According to the standard bacteriological procedures, the microbial culture and identification were performed with the BectecFX200 (BD) and Microbial mass spectrometry system (MALDI-TOFMS, BRUKER, Germany). The minimum inhibitory concentration (MIC) values were interpreted with phonexTMM50 (BD) according to the breakpoint set by the Clinical and Laboratory Standards Institute (2019).

### Statistical analysis

The statistical analysis was performed using R software (Version 3.6.1), SPSS software (Version 24.0), and Excel software (Version 2019). R packages for statistical analysis include compareGroups. Categorical variables are described as frequencies and percentages. Continuous variables with normal distributions are described as the means and standard deviations; otherwise, they are described as the medians (IQRs). An independent-samples t-test was conducted to compare the means of two normally distributed variables. The Mann–Whitney U-test was conducted to evaluate nonnormally distributed variables. The risk factors for three-month mortality in patients with early CRKP infection after LT were identified by univariate and multivariate logistic regression analyses using odds ratios (ORs) and 95% confidence intervals (CIs). COX multivariate regression analysis was conducted to evaluate the difference in the 3-months survival curves between patients with and without CRKP infections. P<0.05 was considered statistically significant.

## Results

### Demographic information of bacteria-infected patients

In recruited 272 LT recipients, sixty-two patients (22.8%) were recorded bacteria infection within 3-months post LT (**Table 1**). The total prevalence of early bacteria infections was 22.8% (62/272), and the mortality was 14.7% (40/272). In bacterial infections, the single-site infection was 35.5% (22/62), and the multiple infections were 64.5% (40/62). The CRKP infection rate in mortality patients (30.0%, 12/19) was significantly higher than survival patients (3.02%, 7/19). Liver failure was the most pre-operation disease in mortality patients (45.0%, 18/40), and HCC was the primary pre-operation disease in survival patients (40.5%, 94/232).

**Table 1:** Clinical information of liver transplant recipients.

	Liver Transplant		p
	Death (40, 14.7%)	Survival (232, 85.3%)	
Mean age(years)	52.4 ± 13.8	51.8 ± 9.81	0.806
Sex			0.06
Female	13 (32.5%)	42 (18.1%)	
Male	27 (67.5%)	190 (81.9%)	
Types of infection			<0.001
No infection	4 (10.0%)	206 (88.80%)	
Blood	1 (2.50%)	1 (0.43%)	
Enterocoelia	3 (7.50%)	6 (2.59%)	
Lung	8 (20.00%)	3 (1.29%)	
Multiple infections	24 (60.00%)	16 (6.90%)	
CRKP infection	12 (30.00%)	7 (3.02%)	<0.001
Pre-operation disease			0.236
ALD	4 (10.0%)	27 (11.6%)	
CHB	0 (0.00%)	1 (0.43%)	
HCC	10 (25.0%)	94 (40.5%)	
LC	5 (12.5%)	32 (13.8%)	
LF	18 (45.0%)	70 (30.2%)	
other	3 (7.50%)	8 (3.45%)	

### Clinical specimens and composition of bacteria pathogens

Bacteria pathogens were cultured and identified from 188 clinical samples (**Table 2**). The top three were sputum (50, 26.6%), ascites (49, 26.1%), and blood (25, 13.3%). In 2019, the top three clinical specimens were sputum (8, 30.8%), ascites (8, 30.8%), and blood (5, 19.2%). The top three clinical specimens in 2020 were sputum (16, 32.0%), ascites (14, 28.0%), and blood (5, 10.0%). The top three infection sites in 2021 were ascites (27, 24.1%), sputum (26, 23.2%), and bloodstream (15, 13.4%).

**Table 2:** Clinical specimens in which bacteria pathogens detected.

Samples	2019	2020	2021	Total
Sputum	8 (30.8%)	16 (32.0%)	26 (23.2%)	50 (26.6%)
Ascites	8 (30.8%)	14 (28.0%)	27 (24.1%)	49 (26.1%)
Blood	5 (19.2%)	5 (10.0%)	15 (13.4%)	25 (13.3%)
Organ preservation solution	0 (0.0%)	7 (14.0%)	10 (8.9%)	17 (9.0%)
Bile	1 (3.8%)	2 (4.0%)	9 (8.0%)	12 (6.4%)
Urine	1 (3.8%)	0 (0.0%)	7 (6.3%)	8 (4.3%)
Incisional secretion	1 (3.8%)	1 (2.0%)	4 (3.6%)	6 (3.2%)
Others	2 (7.7%)	4 (8.0%)	12 (10.7%)	18 (9.6%)
Total	26	50	112	188

A total of 163 strains of pathogenic bacteria were isolated from bacteria infection patients (**Table 3**). The top three were *K. pneumoniae* (30, 18.4%), *A. baumannii* (26, 16.0%), and *E. faecium* (26 strains, 16.0%). In 2019, *K. pneumoniae* (7, 24.1%) was the most, followed by *A. baumannii* (4, 13.8%) and *E. faecium* (4, 13.8%). *E. faecium* (9, 18.0%), *A. baumannii* (8, 16.0%), and *K. pneumoniae* (6, 12.0%) were the top 3 pathogenic bacteria in 2020. In 2021, the top three pathogenic bacteria were *K. pneumoniae* (17, 20.2%), *A. baumannii* (14, 16.7%), and *E. faecium* (13, 15.5%).

**Table 3:** Composition of bacteria pathogens.

Bacteria	2019	2020	2021	Total
*Klebsiella pneumoniae*	7 (24.1%)	6 (12.0%)	17 (20.2%)	30 (18.4%)
*Acinetobacter baumannii*	4 (13.8%)	8 (16.0%)	14 (16.7%)	26 (16.0%)
*Enterococcus faecium*	4 (13.8%)	9 (18.0%)	13 (15.5%)	26 (16.0%)
*Pseudomonas aeruginosa*	1 (3.4%)	5 (10.0%)	7 (8.3%)	13 (8.0%)
*Stenotrophomonas maltophilia*	2 (6.9%)	4 (8.0%)	6 (7.1%)	12 (7.4%)
*Staphylococcus aureus*	2 (6.9%)	3 (6.0%)	6 (7.1%)	11 (6.7%)
Others	9 (31.0%)	15 (30.0%)	21 (25.0%)	45 (27.6%)
Total	29	50	84	163

The CRKP infection (meropenem resistance) was gradually increased among 2019 (2/7, 28.6%), 2020 (4/6, 66.7%), and 2021(13/17, 76.5%). CRKP showed high resistance to penicillins, cephalosporins, and carbapenems. CRKP retains sensitivity to aminoglycoside, chloramphenicol, and tetracycline (**Table 4**).

**Table 4:** Antimicrobial susceptibility of isolates from patients with carbapenem-resistant klebsiella pneumoniae infection.

Antibiotic	Susceptible (%)
Amikacin	36.8%
Acheomycin	57.9%
Chloramphenicol	57.9%
Gentamycin	63.2%
Amoxicillin/clavulanic acid	94.7%
Ceftazidime	94.7%
Piperacillin/tazobactam	94.7%
Aztreonam	100.0%
Meropenem	100.0%
Ampicillin/sulbactam	100.0%
Cefazolin	100.0%
Cefepime	100.0%

### Analysis of mortality and the risk factors to the mortality of LT recipients

Of the 62 recipients with early infections, twenty-eight patients (45.2%) were dead within 3-months post LT. In mortality patients, four patients (14.3%) were with DM, thirteen (46.4%) were with HTA, eleven (39.3%) were with POI, seven (25.0%) were with HCC, thirteen (46.4%) were with LF. In types of infection, eighteen patients (64.3%) were dead with multifocal infection, six (21.4%) were dead from lung infection, and twelve (42.9%) were dead from CRKP infection. The median length of MV was 10.0 days (IQR:3.00, 18.00) in dead patients and 3.00 days (IQR:1.25, 6.75) in survival patients. The length of ICU stay in dead patients (Median:16.00, IQR:9.00;28.50) was longer than survival (Median:9.50, IQR:4.25;23.80).

Univariate logistic regression analysis showed that the potential risk factors for mortality within 3-months after LT were age, CRKP infection, and the length of MV. Multivariate logistic regression analysis showed that age (OR=1.126, 95% CI: 1.009~1.257; P=0.034), CRKP infection (OR=18.240, 95% CI: 2.206~150.842; P=0.007), and the length of MV (OR=1.206, 95% CI:1.039~1.401; P=0.014) were independent risk factors for 3-months mortality post LT (**Table 5**).

**Table 5:** Univariate and multivariate analysis of risk factors for 3-months mortality in liver transplant recipients with early bacteria infection.

	Bacteria infection LT recipients		Univariate analysis		Multivariate analysis	
Variable	Death (28)	Survival (34)	OR (95%CI)	*p*	OR (95%CI)	*p*
Sex, Male (n,%)	20 (71.4%)	28 (82.4%)	0.536 (0.161-1.786)	0.310	1.153 (0.154-8.638)	0.89
Mean age (years)	56.7 ± 9.59	50.8 ± 10.3	**1.064 (1.006-1.126)**	**0.030**	**1.126 (1.009-1.257)**	**0.034**
**Pre-operation disease**						
DM (n,%)	4 (14.3%)	4 (11.8%)	1.25 (0.283-5.525)	0.769	0.774 (0.09-6.624)	0.815
HTA (n,%)	13 (46.4%)	8 (23.5%)	2.817 (0.951-8.345)	0.062	1.6 (0.272-9.425)	0.604
POI (n,%)	11 (39.3%)	17 (50.0%)	0.647 (0.235-1.783)	0.400	0.196 (0.028-1.366)	0.100
HCC (n,%)	7 (25.0%)	8 (23.5%)	1.083 (0.338-3.477)	0.893	0.497 (0.069-3.587)	0.488
LF (n,%)	13 (46.4%)	19 (55.9%)	0.684 (0.25-1.869)	0.459	1.253 (0.208-7.566)	0.806
**Types of infection**						
Multifocal infection (n,%)	18 (64.3%)	21 (61.8%)	1.114 (0.395-3.144)	0.838	2.503 (0.297-21.134)	0.399
Lung infection (n,%)	6 (21.4%)	5 (14.7%)	1.582 (0.427-5.861)	0.493	8.325 (0.519-133.567)	0.134
CRKP infection (n,%)	12 (42.9%)	7 (20.6%)	**2.893 (0.945-8.854)**	**0.063**	**18.240 (2.206-150.842)**	**0.007**
MV (days)	10.00 (3.00;18.00)	3.00 (1.25;6.75)	**1.139 (1.044-1.243)**	**0.003**	**1.206 (1.039-1.401)**	**0.014**
Length of ICU stay (days)	16.00 (9.00;28.50)	9.50 (4.25;23.80)	1.026 (0.991-1.062)	0.154	0.961 (0.908-1.017)	0.167

DM, diabetes mellitus; HTA, hypertension; POI, pre-operation infection; HCC, hepatocellular carcinoma; LF, liver failure; MV, mechanical ventilation. Bold values indicated that these variables were significant in univariate (p<0.1) and multivariate analysis (p<0.05).

### Analysis of risk factors for CRKP infection post LT

Of the 62 bacteria-infected LT recipients, nineteen patients (30.6%) were with CRKP infection, and twelve patients (63.2%) were dead (**Table 6**). In 19 CRKP-infected LT recipients, two patients were with DM in pre-operation, seven were with HTA, ten were with POI, six were with HCC, and eleven were with LF. The length of MV in CRKP infection patients was 3.00 days (IQR:2.00, 17.0), and the length of ICU stay was 6.00 days (IQR:2.00, 10.0). The multivariate logistic regression analysis showed that the length of ICU stay (OR=1.067, 95%CI: 1.015~1.122; P=0.011), POI (OR=6.733, 95%CI: 1.160-39.088; P=0.034), and HCC (OR=26.772, 95%CI: 1.747-410.187; P=0.018) were the independent risk factors for the CRKP infection.

**Table 6:** Univariate and multivariate analysis of the risk of carbapenem-resistant klebsiella pneumoniae infection in liver transplant recipients.

	CRKP infection		Univariate analysis		Multivariate analysis	
Variable	Yes (19)	No (43)	OR (95%CI)	*p*	OR (95%CI)	*p*
Sex, Male (n,%)	13 (68.4%)	35 (81.4%)	0.495 (0.144-1.703)	0.265	0.177 (0.029-1.073)	0.060
Mean age (years)	52.6 (10.6)	53.8 (10.3)	0.988 (0.938-1.042)	0.662	0.961 (0.894-1.032)	0.275
DM (n,%)	2 (10.5%)	6 (14.0%)	0.725 (0.133-3.972)	0.711	1.071 (0.107-10.715)	0.954
HTA (n,%)	7 (36.8%)	14 (32.6%)	1.208 (0.391-3.739)	0.743	1.737 (0.365-8.271)	0.488
POI (n,%)	10 (52.6%)	18 (41.9%)	1.543 (0.521-4.569)	0.433	**6.733 (1.160-39.088)**	**0.034**
HCC (n,%)	6 (31.6%)	9 (20.9%)	1.744 (0.517-5.875)	0.370	**26.772 (1.747-410.187)**	**0.018**
LF (n,%)	11 (57.9%)	21 (48.8%)	1.44 (0.485-4.282)	0.511	5.035 (0.597-42.47)	0.137
MV (days)	3.00 (2.00;17.0)	6.00 (2.00;10.0)	1.045 (0.99-1.104)	0.113	1.008 (0.935-1.086)	0.841
Length of ICU stay (days)	25.00 (9.50;36.0)	10.00 (5.00;18.0)	**1.056 (1.015-1.098)**	**0.007**	**1.067 (1.015-1.122)**	**0.011**

DM, diabetes mellitus; HTA, hypertension; POI, pre-operation infection; HCC, hepatocellular carcinoma; LF, liver failure; MV, mechanical ventilation. Bold values indicated that these variables were significant in univariate (p<0.1) and multivariate analysis (p<0.05).

**Figure 1:**
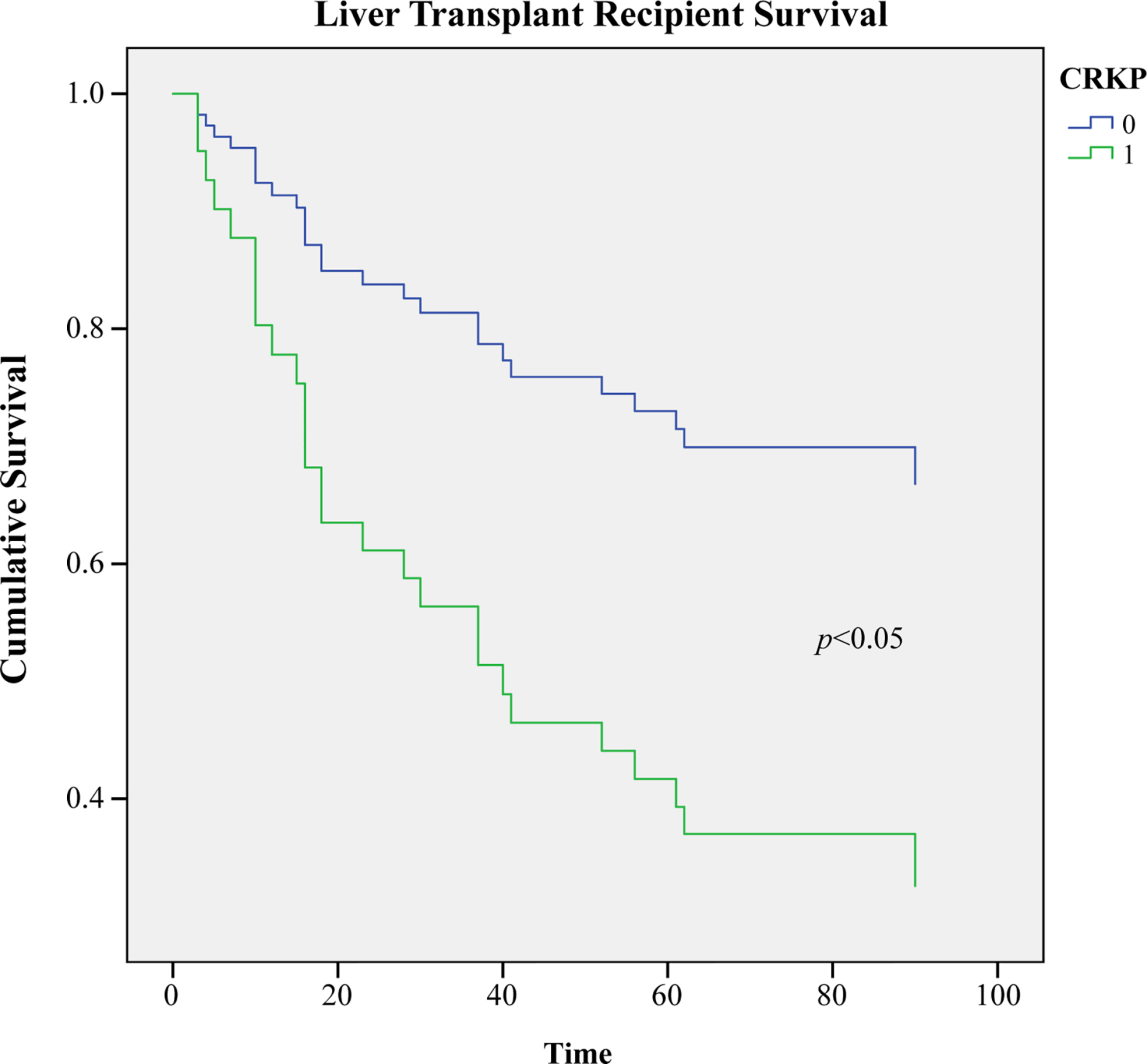
Comparison of the 3-months survival rate of infected and uninfected CRKP among early Liver Transplant recipients (*p*<0.05).

According to COX multivariate regression analysis, the overall survival rate of CRKP infected patients was significantly lower than that without CRKP infection (*p*<0.05, **Figure 1**).

## Discussion

LT recipients are prone to opportunistic infection and transmission of drug-resistant bacteria, and the influencing factors include malnutrition, surgical attack, and immunosuppressive treatments. Most bacterial infections occur within the early stage after transplantation (19%-33%) (Kim, 2014), with a mortality range from 24% to 36% (Kim et al., 2009). In LT recipients, the mortality caused by GNB infection (39.3%) was significantly higher than GPB infection (4.8%) (Chen et al., 2020). It is estimated that 10%-20% of solid organ transplant recipients are infected with multidrug-resistant GNB, in which carbapenem-resistant *Klebsiella pneumoniae* (CRKP) is the most lethal (Barchiesi et al., 2016; Bias et al., 2018). Our survey showed that the bacteria infection rate was 22.9%, and the mortality was 13.09% in the first 3-months post-LT between 2019 with 2021. Bacterial infection remains the leading cause of early death in LT recipients. The detected specimens mainly include sputum (26.6%), ascites (26.1%), and blood (13.3%), which reflects the infection sites are mainly lung, abdominal cavity, and blood. GNB is the primary pathogen in which *K. pneumoniae* was the most (18.4%). The *K. pneumoniae* drug resistance rate of carbapenems reached 63.3%. CRKP infections increased year by year among 2019 (28.6%), 2020 (66.7%), and 2021(76.5%). CRKP infections account for 30% of deaths caused by bacteria infection. CRKP infection is still the fatal factor in the early death of LT recipients, and the infection rate is increasing year by year. Based on the epidemic, effective prevention and control strategies for CRKP infection in LT recipients are still essential.

CRKP infection (OR=18.240), length of MV (OR=1.206), and age (OR=1.126) were independent risk factors for the death of bacteria-infected LT recipients (p<0.05). CRKP infection, long MV time, and older LT recipients were closely associated with bacterial infection death. Previous studies have shown that CRKP infection is closely associated with SOT recipient death, with the mortality of 40~75% (Bias et al., 2018; Wang et al., 2020). Our survey showed that twelve (63.2%) cases were dead in 19 CRKP-infected recipients. In kidney transplant recipients, the length of MV (>48h) was an independent risk factor for recipient infection death and CRKP infection (Zhang et al., 2021). Some case-control studies also showed that longer MV length, longer ICU length, and elder age were independent risk factors for CRKP infection mortality (Jiao et al., 2015; Akgul et al., 2016; Chen et al., 2022). In our study, the COX multivariate regression analysis showed that the 3-months survival rate post-LT in the CRKP-infected group was significantly lower than that without CRKP infection. Therefore, effective control of CRKP infection contributes to a favorable prognosis for LT recipients.

Our study showed that the length of ICU (OR=1.067), pre-operation infection (OR=6.733), and HCC (OR=26.772) were the independent risk factors for CRKP infection (*p*<0.05). Previous studies have shown that clear the pre-operation infections can effectively prevent the occurrence of post-operation adverse events (Ahmed and Keeffe, 2007; Kim, 2014; Pereira et al., 2015). Other studies shown that pre-operation MELD score and liver failure are the independent risk factors for CRKP infections in LT recipients (Barchiesi et al., 2016). Our analysis showed that HCC was associated with a higher risk of CRKP infection, which is consistent with the results obtained in previous studies and the mechanism may the tumor immunosuppression (Pereira et al., 2015). Longer ICU stay is another risk factor for CRKP infection in LT recipients. Some studies have shown that ICU patients has a higher intestinal and nasopharyngeal colonization of CRKP (Qin et al., 2020). Thus, decreasing the ICU stay post-LT are significant for CRKP infection avoidance in LT recipients. The meta-analysis also showed that pre-operation MELD score, preoperative primary disease, bleeding during the operation (Giannella et al., 2015; Mazza et al., 2017; Pagani et al., 2021), Roux-en-Y biliary choledochojejunostomy, and bile leak (Pereira et al., 2015) are the risk factors for CRKP infections in LT recipients. Therefore, effective strategies to control pre-operative, intra-operative, and post-operative risk factors can significantly reduce CRKP infection in LT recipients.

This study has some limitations. First, this retrospective analysis represents the regional prevalence of bacterial and CRKP infection in LT recipients, which needs more multicenter studies to monitor the overall epidemic. Second, more work is needed to carry out CRKP strains’ molecular characteristics and antibiotic resistance mechanism of CRKP strains.

## Conclusion

In summary, our research investigated pathogens’ composition, the CRKP epidemic, and risk factors for mortality and CRKP infection of LT recipients in 3-months post-LT. The early stage of mortality in LT recipients is positively associated with the CRKP infection, the longer length of MV, and elder age. The length of ICU, HCC, and POI are the independent risk factors for CRKP infection in LT recipients. Therefore, effective strategies for pre-operation disease control and length of MV and ICU reduction could help for fatal CRKP infection in LT recipients.

## Data availability statement

The raw data supporting the conclusions of this article will be made available by the authors, without undue reservation.

## Ethics statement

The studies involving human participants were reviewed and approved by ethics committee of Beijing Youan Hospital. Written informed consent for participation was not required for this study in accordance with the national legislation and the institutional requirements.

## Author contributions

NL, YY, JL, and DC, study design, statistical analysis, data interpretation, manuscript preparation, and literature search. YD, MC, FD, XD, clinical laboratory data collection, and interpretation. GY, WL, GL, clinical data collection and interpretation. All authors contributed to the article and approved the submitted version.

## Funding

This work was supported by Beijing Municipal Education Commission Science and Technology Program (KZ202010025037), Beijing Talents Foundation (2018000021469G287), Project of Beijing Municipal Science and Technology Commission (Z201100007920011, Z221100007922009), Capital Health Development Scientific Research Special Public Health Project (2021-1G-4301, 2021-1G-4302), Youan Liver Disease AIDS Fund (BJYAYY-GG2019-04).

## Acknowledgments

The authors thank the colleagues of the Scientific Research Department of the hospital for their support.

## Conflict of interest

The authors declare that the research was conducted in the absence of any commercial or financial relationships that could be construed as a potential conflict of interest.

## Publisher’s note

All claims expressed in this article are solely those of the authors and do not necessarily represent those of their affiliated organizations, or those of the publisher, the editors and the reviewers. Any product that may be evaluated in this article, or claim that may be made by its manufacturer, is not guaranteed or endorsed by the publisher.

## Abbreviations

LT, Liver transplantation; GNB, Gram-negative bacteria; GPB, Gram-positive bacteria; KP, Klebsiella pneumoniae; CRKP, Carbapenemresistant Klebsiella pneumoniae; MDR, Multi-drug-resistant; DM, Diabetes mellitus; HTA, Hypertension; POI, Pre-operation infection; HCC, Hepatocellular carcinoma; LF, Liver failure; MV, Mechanical ventilation; ICU, Intensive care unit; ORs, Odds ratios; CIs, Confidence intervals.
